# In Vivo Inhibition of Marek’s Disease Virus in Transgenic Chickens Expressing Cas9 and gRNA against ICP4

**DOI:** 10.3390/microorganisms9010164

**Published:** 2021-01-13

**Authors:** Arjun Challagulla, Kristie A. Jenkins, Terri E. O’Neil, Shunning Shi, Kirsten R. Morris, Terry G. Wise, Prasad N. Paradkar, Mark L. Tizard, Timothy J. Doran, Karel A. Schat

**Affiliations:** 1 CSIRO Health and Biosecurity, Australian Centre for Disease Preparedness, Geelong, VIC 3220, Australia; arjun.challagulla@csiro.au (A.C.); Kristie.Jenkins@csiro.au (K.A.J.); Terri.O'Neil@csiro.au (T.E.O.); Shuning.Shi@csiro.au (S.S.); Kirsten.Morris@csiro.au (K.R.M.); Terry.Wise@csiro.au (T.G.W.); Prasad.Paradkar@csiro.au (P.N.P.); Mark.Tizard@csiro.au (M.L.T.); 2 Department of Microbiology and Immunology, College of Veterinary Medicine, Cornell University, Ithaca, NY 14853, USA; kas24@cornell.edu

**Keywords:** Marek’s disease virus, CRISPR/Cas9, ICP4, transgenic chicken, disease resilience

## Abstract

Marek’s disease (MD), caused by MD herpesvirus (MDV), is an economically important disease in chickens. The efficacy of the existing vaccines against evolving virulent stains may become limited and necessitates the development of novel antiviral strategies to protect poultry from MDV strains with increased virulence. The CRISPR/Cas9 system has emerged as a powerful genome editing tool providing an opportunity to develop antiviral strategies for the control of MDV infection. Here, we characterized Tol2 transposon constructs encoding Cas9 and guide RNAs (gRNAs) specific to the immediate early infected-cell polypeptide-4 (ICP4) of MDV. We generated transgenic chickens that constitutively express Cas9 and ICP4-gRNAs (gICP4) and challenged them via intraabdominal injection of MDV-1 Woodlands strain passage-19 (p19). Transgenic chickens expressing both gRNA/Cas9 had a significantly reduced replication of MDV in comparison to either transgenic Cas9-only or the wild-type (WT) chickens. We further confirmed that the designed gRNAs exhibited sequence-specific virus interference in transgenic chicken embryo fibroblast (CEF) expressing Cas9/gICP4 when infected with MDV but not with herpesvirus of turkeys (HVT). These results suggest that CRISPR/Cas9 can be used as an antiviral approach to control MDV infection in chickens, allowing HVT to be used as a vector for recombinant vaccines.

## 1. Introduction

Marek’s disease (MD), a lymphoproliferative disorder in chickens, is caused by Marek’s disease herpesvirus (MDV), a strictly cell-associated herpesvirus. MD causes an estimated economic loss of USD 1 billion to the global poultry industry [1]. MDV belongs to the genus *Mardivirus* of the subfamily *Alphaherpesvirinae* in the *Herpesviridae* family [2]. MDV includes two serotypes, with all pathogenic strains belonging to serotype 1, while non-oncogenic strains (e.g., SB-1 [3]) belong to serotype 2. In addition, the related herpesvirus of turkeys (HVT) [4] is often referred to as serotype 3 [2]. MDV infection is initiated by inhalation of infectious cell-free virions into the respiratory tract which is then transported to lymphoid organs. MDV replicates efficiently in B cells and infects activated T lymphocytes, primarily in CD4+ cells where it establishes latent infections. Infected T cells transport the virus to the feather follicle epithelium where the cell-free infectious virus is shed into the environment. Depending on the genetic resistance to MD and vaccination status, tumors may develop in susceptible chickens [5,6].

The development of the first-generation vaccines including HVT [7] and CVI988, a low-pathogenic, attenuated serotype 1 strain [8], greatly reduced MD losses. However, since the introduction of vaccines in the early 1970s, MDV has increased in virulence, and currently, three pathotypes are recognized: virulent, very virulent (vv) and vv+ [9]. Thus far, CVI988, CVI988+HVT and HVT+SB-1 are still providing excellent protection. Gimeno [10] discussed the need to improve genetic resistance by transgenesis, perhaps using RNA interference when the pathogenicity of MDV increases once more and CVI988 no longer provides protection. The use of targeted nuclease systems such as CRISPR/Cas9 that can directly act on viral DNA may present a possible new strategy to control MDV.

The genome of MDV consists of dsDNA with an approximate size of ~177 kb, encoding more than ~100 proteins [11]. Selective disruption of one or more essential genes can lead to abrogation of viral replication in host cells. Previous studies using RNA interference (RNAi)-mediated knockdown of infected-cell polypeptide-4 (ICP4) and glycoprotein B (gB) genes showed their essential role in MDV replication as determined by the reduction in viral titers and plaque size in vitro [12] and in vivo [13,14]. However, small interfering RNAs (siRNAs) target mRNA transcripts and do not target viral DNA in the host cells. Moreover, stable expression of short hairpin RNA (shRNA) is prone to cellular toxicity by overloading the native miRNA pathway with shRNAs [15,16]. The CRISPR/Cas9 system is an adaptive innate immune system that provides resistance to microbes by targeting the nucleic acid of invading bacteriophages in a sequence-specific manner [17]. Due to the specificity of the robust DNA cleavage activity, the CRISPR/Cas9 system has emerged as a powerful genome editing tool, providing an opportunity to develop novel strategies to control DNA virus infections in eukaryotes. By co-expressing plasmids encoding guide (g)RNAs and Cas9 in cells, targeted double-strand breaks (DSBs) can be achieved at any given sequence in cellular genomes [18,19]. CRISPR/Cas9 has been shown to be effective at inhibiting herpes simplex virus (HSV) [20] and hepatitis B virus [21] as well as the dsDNA form of human immunodeficiency virus [22]. Recently, CRISPR/Cas9-mediated in vitro inhibition of MDV replication of a very virulent strain of MDV, the RB-1B strain, was demonstrated by targeting essential genes for its replication [23].

In the present study, we generated a transgenic chicken line that stably express functional ICP4 gRNAs and Cas9 and challenged these chickens with MDV. Virus isolation rates were significantly lower in transgenic chickens expressing Cas9/gICP4 than in transgenic Cas9 or wild-type (WT) chickens. This strategy may provide a novel and effective approach to facilitate the control of MDV in chickens.

## 2. Materials and Methods

### 2.1. Animal Husbandry

Maintenance and breeding of experimental chickens (Hy-Line brown) was conducted at the CSIRO Werribee Animal Facility under Biosecurity containment level 2 (BC2) conditions. All in vivo MDV experiments were conducted at the CSIRO Australian Centre for Disease Preparedness (ACDP). All protocols used to generate transgenic chickens and in vivo infection work were performed following the guidelines of the Australian code for the care and use of animals for scientific purposes. The Animal Ethics Committee (AEC) of the CSIRO ACDP approved all experiments described in this study (AEC approval numbers: 1849, 1886, 1920 and 1934).

### 2.2. Cell Culture

Primary chicken embryo fibroblast (CEF) cultures were prepared from 9- to 10-day-old embryos obtained from wild-type (WT) or transgenic Hy-Line brown chickens. The chicken fibroblast cell line DF1 [24] (American Type Culture Collection number: CRL-12203) and CEF cultures were maintained in Dulbecco’s Modified Eagle’s Medium (DMEM) supplemented with 10% fetal calf serum (FCS), 2 mM L-glutamine, 10 mM Hepes, 1.5% (*w*/*v*) sodium bicarbonate, 100 U/mL penicillin and 100 μg/mL streptomycin. All cells were incubated at 37 °C but shifted to 38.5 °C for MDV infected CEF cultures in a 5% CO_2_/95% air atmosphere.

### 2.3. Viruses

The MDV vaccine strains CVI988 [8] and HVT FC126 [4] were purchased from Bioproperties (Australia) and propagated in CEF. The very virulent (vv) MDV Woodland strain passage-18 (p18) [25,26] was a gift from Professor Tim Mohany at the University of Queensland, Australia. The MDV Woodland stock (p19) used for in vivo experiments was propagated in CEF. The virus stocks were stored in liquid nitrogen and titrations were performed in CEF to determine the PFU/mL.

### 2.4. Design of gRNAs

The gRNAs targeting the ICP4 gene were designed using the https://zlab.bio/guide-design-resources website by providing specific sequences for the proximal, middle and distal locations of ICP4 identified in Figure 2a as Pair-1, Pair-2 and Pair-3, respectively. Specifically, Pair-1 (gRNA1 and gRNA2) covers nucleotides –168 (upstream to ATG, ICP4 promoter region) to 78 from the ATG start codon; Pair-2 (gRNA3 and gRNA4) covers nucleotides 3275 to 3525 from the ATG start codon and Pair-3 (gRNA5 and gRNA6) covers nucleotides 6174 to 6394 and 6411 to 6528 from the start codon ATG, respectively. The gRNAs were selected based on the highest on-target score. Using BLAST analysis, it was determined that all 6 designed MDV ICP4 gRNAs (hereafter referred to as gICP4) do not target sequences in the chicken genome. The gRNA sequences are provided in Table 1. The six designed gRNAs were cloned into CRISPR/Cas9 constructs using synthesized oligonucleotides. The sequences are listed in Supplementary Table S1.

### 2.5. Plasmids

The CRISPR expression plasmids pX458 (plasmid No. 48138) and pX333 (Plasmid No. 64073) were obtained from Addgene (USA). The plasmid pX458 contains a single gRNA cloning site (BbsI) while pX333 contains two gRNA cloning sites (BbsI and BsaI). The ICP4-specific gRNAs were cloned into the vector by following the protocol described by Ran et al. [27] and verified by sequencing. The gRNA targeting the green fluorescent protein (GFP) gene was previously described [19] and was purchased from Addgene (USA) (Plasmid #41820). The miniTol2 vector used in this study was previously described [28] and was a gift from Professor S. C. Ekker, Mayo Clinic Cancer Center, Minnesota, USA.

### 2.6. Construction of gRNA-DsRed and Cas9-GFP

The backbone vector to clone the high-fidelity Cas9 (Cas9-HF1) and GFP (hereafter referred to as Cas9-GFP) or gICP4-DsRed was a miniTol2 transposon construct, previously described [29]. We constructed 3 Tol2 vectors encoding Cas9 and gRNAs targeting the ICP4 or non-specific (NS) control (Figure S1). Vector 1 contained the Cas9 gene and the GFP gene that was previously published [30]. Vector 2 consisted of ICP4 gRNAs (gICP4#1, gICP4#4 and gICP4#6), each under the control of the human U6 (hU6) promoter to drive expression of the three gRNAs, and the DsRed gene under the control of the cytomegalovirus immediate early (CMV-IE) promoter. Vector 3 was identical to vector 2, except that we replaced the ICP4 gRNAs with 3 non-specific (NS) gRNAs (gNS), each under the control of hU6, and DsRed under the control of the CMV-IE promoter serving as a negative control. Using BLAST analysis, it was determined that the three gNS sequences (~20 bp of either a BbsI or BsaI restriction site sequence) used in the study do not target ICP4 or the chicken genome.

The Tol2-GFP-Cas9 (vector 1) contains CMV-Cas9-HF1 and CAGG-GFP inserted between the two inverted terminal repeats of Tol2 (Figure S1). To generate a Tol2 transposon vector with three ICP4 gRNAs (gICP4#1, gICP4#4 and gICP4#6) and DsRed coding sequences, we performed the cloning in five steps. In step 1, gICP4#1 was cloned into the BbsI site of pX458 while gRNA4 and gRNA6 were separately cloned into the BbsI and BsaI sites of the pX333 vector following the gRNA cloning protocol described by Ran et al. [27]. In step 2, we PCR-amplified the single transcription unit which includes U6-gICP4#1 in the pX458 vector using PCR-pX458-Fwd and PCR-pX458-Rev primers with introduced SalI and XhoI sites and cloned the amplicon into the pGEM-T Easy vector. Similarly, the two gRNA transcription units which include the U6-gICP4#4+U6-gICP4#6 region in the pX333 vector were amplified using PCR-pX333-Fwd and PCR-pX333-Rev with introduced SalI site and XhoI sites and cloned into the pGEM-T Easy vector. Both amplicons were verified by sequencing. In step 3, the combined U6-gICP4#4+U6-gICP4#6 fragment in the pGEM-T Easy vector was excised by digesting with SalI and XhoI and cloned into the XhoI site alongside gICP4#1 in the pGEM-T Easy vector to generate three transcription units in tandem (i.e., U6-gICP4#1+U6-gICP4#4+U6-gICP4#6). In step 4, the fragment containing three transcription units in pGEM was digested and cloned directionally into the EcoRI site of the base miniTol2 vector. In step 5, The CMV-DsRed fragment was amplified from the pDsRed-Express2-C1 vector (Catalogue No. 632538, Clontech) using DsRed-Fwd and DsRed-Rev primers and cloned into the SpeI and NotI sites of the miniTol2 vector adjacent to three U6-gICP4 transcription units to generate the final Tol2-gICP4#1-#4-#6+DsRed vector (from now on referred to as Tol2-gICP4-DsRed) (vector 2, Figure S1). To generate a non-specific (NS) control gRNA plasmid (vector 3), we used the primer pair PCR-pX333-Fwd and PCR-pX333-Rev to amplify pX333 and the primer pair PCR-pX458-Fwd and PCR-pX458-Rev to amplify pX458 with the non-specific (NS) 20-bp sequence consisting of BbsI and BsaI restriction enzyme sequences. We subsequently cloned three NS gRNAs transcription units in tandem using the XhoI and SalI cloning strategy described above to generate Tol2-NS-gRNA-DsRed (from now on referred to as Tol2-gNS-DsRed). The sequences of all primers used in this study are listed in Supplementary Table S1.

### 2.7. Characterization of gRNAs for Targeted Cleavage of ICP4

**Figure 1:**
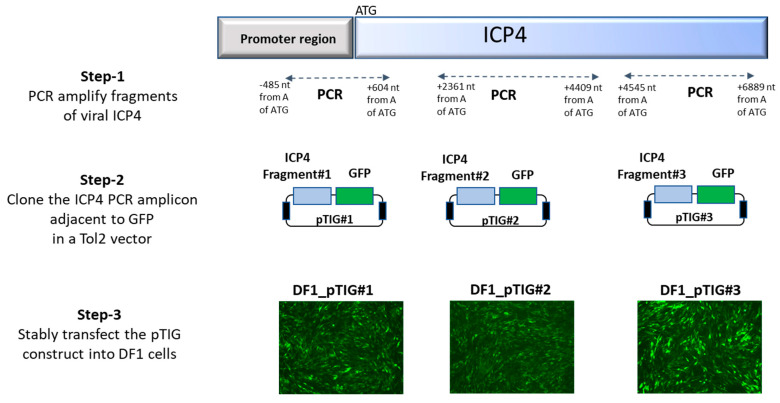
Schematic depiction of generating DF1 cells carrying the infected-cell polypeptide-4 (ICP4) gene fragments and GFP in a Tol2 transposon vector. Three stable DF1 cells carrying the ICP4 and GFP transgenes (DF1-pTIG#1, DF1-pTIG#2 and DF1-pTIG#3) were sorted after 2 weeks post-transfection.

To examine the effectiveness of the designed gRNAs, we generated DF1 cells stably transfected with ICP4 and GFP transgenes, followed by examination for targeted cleavage by gRNAs. The process of generating transgenic DF1 cells (DF1_pTIG) carrying ICP4 gene fragments and GFP gene is depicted in Figure 1. In step 1, three PCR fragments with gRNA target sites were amplified using primers targeting (a) the proximal region of 1080 bp—position −485 to 605 nt from start codon ATG using primers ICP4-P-Fwd and ICP4-P-Rev; (b) the middle region of 2048 bp—position 2362 to 4410 nt from start codon ATG using primers ICP4-M-Fwd and ICP4-M-Rev; and (c) the distal end region of 2344 bp—position 4546 to 6890 nt from start codon ATG using primers ICP4-D-Fwd and ICP4-D-Rev. In step 2, the amplicons were cloned into the pGEM-T Easy vector, sequenced and excised by EcoRI digestion to insert alongside the GFP gene in a Tol2-GFP vector to generate Tol2_ICP4_GFP (pTIG) constructs pTIG#1, pTIG#2 and pTIG#3, respectively. In step 3, stable DF1 cell lines carrying ICP4 genomic fragments were generated by transfection with 2 ug of the respective pTIG vector (pTIG#1, #2 or #3) and 2 ug of the pTransposase vector (test) or an empty vector (control). Transfected DF1 cells were passaged and followed by sorting for GFP expression at 2 weeks post-transfection.

To validate if the designed gRNAs were able to create targeted double-strand breaks (DSBs) on ICP4 fragments, DF1-pTIG (#1, #2 or #3) cells were seeded at a density of 3 × 10^6^ cells/well in a six-well plate 24 h prior to transfection. Cells were transfected using Lipofectamine^®^ 2000 (L2000) (Thermo Fisher Scientific, Walthan, MA, USA) transfection reagent with 1.5 µg gRNA-GFP and 2.5 µg of pX333 containing gICP4#1-gICP4#2, gICP4#3-gICP4#, gICP4#5-gICP4#6 or empty pX333. The ICP4-targeted cells were separated from the non-transfected cells based on the absence of fluorescence in the non-GFP DF1-pTIG cells using fluorescence-activated cell sorting (FACS). To determine the cleavage of the ICP4 gene in DF1-pTIG cells, PCR screening was performed using primers spanning across the targeted ICP4 region (ICP4-#1-Fwd and ICP4-#1-Rev, ICP4-#2-Fwd and ICP4-#2-Rev and ICP4-#3-Fwd and ICP4-#3-Rev) on corresponding transfected DF1 cells. PCR assays were performed using 12.5 µL GoTaq^®^ Green Master Mix (Catalogue No. M7122, Promega), 1 µL forward primer (10 µM), 1 µL reverse primer (10 µM), template DNA (~50 ng per reaction) and the required amount of DNase-free water to make the final volume of 25 µL per reaction. A thermocycler (Eppendorf) was used with the following cycling parameters: one cycle at 95 °C for 10 min; 35 cycles at 95 °C for 30 sec, 53–57 °C for 30 s and 72 °C for 1 min; and a final extension step at 72 °C for 5 min. The extension temperature varied from 55 to 58 °C depending on the melting temperature of the primers. The wild-type and truncated PCR amplicons were resolved by gel electrophoresis. The CRISPR/Cas9-mediated targeted cleavage by respective gRNAs was verified by sequencing.

### 2.8. Flow Cytometry and Cell Sorting

Transfected cells were harvested, resuspended in FACS buffer (5% FCS in PBSA) and analyzed using BD FACSAria II (BD Biosciences, San Jose, CA, USA) equipped with 530 nm and 561 nm lasers with 530/30 (GFP) and 582/15 (DsRed) emission filters. From each sample, 30,000 cells were acquired and gated for the analysis using standard forward/side scatter (FSC/SSC) parameters.

### 2.9. Inhibition of CVI988 in CEF Transfected with Plasmids Encoding gRNAs and Cas9

CEF were seeded at a density 3 × 10^6^ cells/well in 6-well plates and transfected using L2000 reagent with 3 µg Tol2-Cas9-GFP (vector 1) and 2 µg Tol2-gICP4-DsRed (vector 2) or Tol2-gNS-DsRed (vector 3) (Figure S1). Transfected CEF were sorted using FACS to isolate the positive cells, which were seeded into 96- or 24-well plates in duplicate and infected with 10 plaque-forming units (PFU) of CVI988. Three days post-infection, the cells were passaged onto freshly transfected CEF and incubated for 3 days. This procedure was performed for 4 passages and PFU were enumerated at 5 days post-infection (dpi) in p4. 

### 2.10. Generation and Breeding of Transgenic Chickens Expressing gICP4-DsRed or Cas9-GFP

The origin of the transgenic Cas9-positive hen used in this study was previously published [30]. This hen harbors a single copy of a Cas9-GFP transgene in chromosome 12 of the genome. To expand this line, further breeding of the G1 Cas9 hen to a WT rooster was performed, and the offspring were raised to sexual maturity. We generated Cas9 roosters and hens for downstream breeding of chicks for in vivo MDV challenge studies.

To introduce the gICP4-DsRed transgene insert into the germline, we performed direct injection of the miniTol2-gICP4-DsRed construct (Figure S1, vector 2) into chicken embryos as previously described by Tyack et al. [29]. Briefly, 2 μg miniTol-gICP4-DsRed and 2 μg pTransposase were complexed with 10 μL Lipofectamine 2000 CD (L2000CD) (Catalogue No. 12566014, Invitrogen) in 80 µL OptiPRO (Catalogue No. 12309019, Thermo Fisher). Approximately 1–2 µL of the L2000CD-DNA complex was injected into the dorsal aorta of stage-14–15 Hamburger Hamilton (HH) chicken embryos. Prior to performing the injections to hatch chicks, we examined whether stable insertion of the gICP4-DsRed transgene caused any toxicity issues in the primordial germ cells (PGCs) of embryonic gonads. For this toxicity assay, gonads from the injected embryos were harvested at stage-40 HH embryos and examined for expression of DsRed under a Leica DMLB fluorescence microscope with a Leica DC 300F digital camera. To generate transgenic chickens carrying the gICP4-DsRed transgene, injected embryos were hatched, and their sex was determined using genomic DNA extracted from the blood of the hatched chicks by following the protocol described by Lambeth et al. [31]. Only male chickens were raised to sexual maturity. The transgenesis rate of gICP4-DsRed transgene in the semen was quantified by qPCR as previously described [29]. To generate G1 transgenic chickens, the G0 roosters with the highest levels of gICP4-DsRed transgene insert in the analyzed samples were mated with 7–10 WT hens. The hatched G1 chicks were screened for the presence of gICP4-DsRed insert by visualizing DsRed expression using DsRed (light source emission Green 470–590) goggles (BLS LTD, Budapest, Hungary). The insertional-site PCR analysis for the Tol2-gICP4-DsRed transgene insert in the genome was performed as previously described by Lambeth et al. [31]. 

### 2.11. Genotyping and Characterization of Cas9/gICP4 CEF

To examine the functionality of Cas9 and gICP4 against MDV, CEF were prepared from individual 10-day-old embryos derived from the breeding of a transgenic Cas9-GFP hen with a G1 gICP4-DsRed rooster. To determine genotypes, genomic DNA from each CEF culture was extracted to perform PCR using primers targeting (a) a ~500-bp long Cas9 transgene using the primers Cas9-Fwd and Cas9-Rev, (b) a ~700-bp long region of gICP4 transgene sequence using the primers gRNA-Scr-Fwd and gRNA-Scr-Rev and (c) a ~450-bp long endogenous gene ovomucoid (OVM) using the primers OVM-Fwd and OVM-Rev. To examine fluorescence, CEF cultures were examined for GFP and DsRed expression using a fluorescence microscope (Leica, Wetzlar, Germany).

### 2.12. Infection of Transgenic CEF Expressing Cas9/gICP4 with MDV and HVT

To examine the efficacy of Cas9/gICP4 against MDV replication, CEF cultures from individual embryos were seeded in duplicate at 2.5 × 10^6^ cells per well in 6-well plates. CEF cultures were infected with 100 PFU of MDV Woodland strain (p19) per culture, and plaques were counted at 5 dpi. To determine sequence-specific targeting of the designed ICP4 gRNAs, WT CEF and Cas9/gICP4-positive CEF were also infected with 100 PFU of HVT and plaques were counted at 5 dpi.

### 2.13. Infection of gICP4/Cas9 Chickens

To generate transgenic chickens for in vivo infection studies, gICP4-DsRed roosters were mated with Cas9 hens, and a single Cas9 male was mated with 4 gICP4-DsRed hens. Offspring were characterized for the presence of Cas9, gICP4 or Cas9/gICP4 transgenes based on the following fluorescence expression pattern: GFP alone (Cas9), DsRed alone (gICP4 alone), GFP+DsRed (Cas9/gICP4) or WT. The GFP and DsRed expression in hatched chicks was visualized using GFsP-5 (long-wavelength blue) or DsRed (light source emission Green 470–590) goggles (BLS LTD, Budapest, Hungary).

To eliminate bias, in vivo trials were performed as single blinded experiments, in which the genotypes of the transgenic chickens were not known to the operators until after the virus isolation assay was conducted. Two experiments were conducted to test the effect on MDV replication. In experiment 1, which consisted of two independent trials (trial 1 and trial 2), and in experiment 2, the WT, Cas9 and Cas9/gICP4 7-day-old chickens were challenged by intraabdominal (ia) inoculation with approximately 5000 PFU of MDV Woodland (p19). All inocula were back-titrated to provide the actual number of PFU. Chickens were humanly euthanized at 6 (trial 1 and trial 2) and 12 (trial 2) dpi in experiment 1 and at 33 dpi in experiment 2, and spleens were collected in phosphate-buffered saline (PBS). Uninoculated control chicks were housed in a separate room. Chickens were monitored twice/day for clinical symptoms during the experiments.

### 2.14. Processing of Splenocytes and Virus Isolation Studies

Spleens from challenged birds were aseptically collected and processed through a 100-µm cell strainer (Catalogue No. CLS431752-50EA, Sigma) to obtain single cell suspensions. Cells were washed with PBS and separated by Ficoll-Hypaque using standard procedures [32]. To determine virus titers, 5 × 10^5^ splenocytes were inoculated in duplicate onto CEF cultures in 6-well plates incubated at 38.5 °C. PFU were enumerated at 5 dpi. Results are shown as number of PFU per 5 × 10^5^ splenocytes.

### 2.15. TaqMan qPCR

The total DNA from splenocytes was extracted using the DNeasy Blood and Tissue Kit (Catalogue No. 69504, Qiagen). A quantitative PCR (qPCR) targeting ICP4 was performed for the quantification of ICP4 DNA. The primers and probes used for the qPCR were designed using Primer Express 3.0 software (Applied Biosystems) and are listed in Supplementary Table S1. These primers and probes do not overlap with the deletion sites targeted by Cas9/gICP4. The endogenous chicken gene, Gallus gallus rho-globin, beta-H globin, beta-A globin, epsilon-globin and olfactory receptor-like protein COR3’beta (COR3’beta) (GenBank: L17432.1) was used as template control, and the details of the primers and probes were previously described by Tyack et al. [29]. The ICP4-specific detection probe used in this study was modified with FAM and TAMRA at the 5′ and 3′ ends, respectively. Primers and probes were obtained from Integrated DNA Technologies (IDT), Australia. The qPCR assays were performed using the StepOnePlus Real-time PCR system machine (Applied Biosystems). Each reaction was comprised of 1.25 μL of each primer (18 μM) and the corresponding probe (5 μM), 2 μL template DNA (50 ng per reaction) and 6.7 μL DNase-free water and TaqMan™ Universal PCR Master Mix (Cat No. 4304437, Thermo Fisher Scientific). The parameters consisted of one cycle at 50 °C for 2 min and at 95 °C for 10 min, 40 cycles at 95 °C for 15 s and 60 °C for 1 min. qPCR data were analyzed using the Delta Ct method [33].

### 2.16. Statistical Analysis

All statistical analyses were carried out using GraphPad Prism 7 software. For CEF experiments, two-tailed, unpaired *t-*tests with the Mann–Whitney test were performed. *p* values less than 0.05 were considered significant. For in vivo infection studies, a nonparametric one-way analysis of variance (ANOVA) and the Kruskal–Wallis test followed by Dunn’s test of multiple comparisons were performed. *p* values less than 0.05 were considered significant.

## 3. Results

### 3.1. Design and Validation of gRNAs Targeting the ICP4 Gene

**Figure 2:**
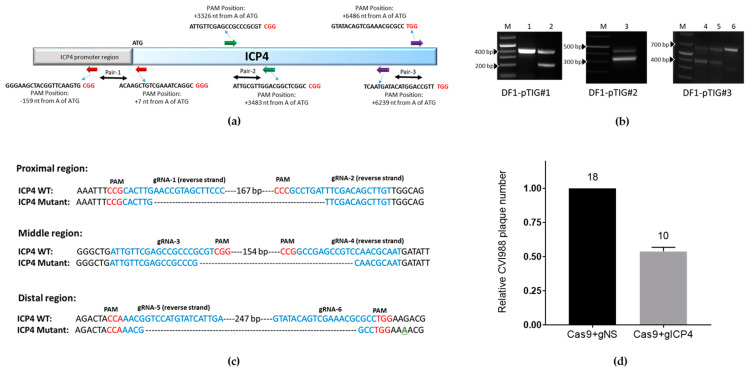
Design and characterization of the guide RNAs (gRNAs) targeting ICP4 gene. (**a**) Schematic representation of six gRNAs designed to target the ICP4 gene. The PAM sequence was highlighted in red color. (**b**) Assessment of gRNAs against ICP4. DF1-pTIG#1, DF1-pTIG#2 and DF1-pTIG#3 cells were co-transfected with GFP-gRNA and a pair of ICP4-gRNAs in pX333 or an empty pX333 vector. Agarose gel electrophoresis of PCR products amplified using primers spanning across the targeted regions, respectively. Expected PCR amplicons: proximal DF1-pTIG#1 (wild-type (WT): ~400 bp; mutant: ~210 bp), middle DF1-pTIG#2 (WT: ~480 bp; mutant: ~300 bp) and distal DF1-pTIG#3 (WT: ~650 bp; mutant: ~400 bp). Targeted deletion of proximal (lane 2), middle (lane 3) and distal regions (lane 4 and 5) of ICP4 gene fragments in pTIG cells by corresponding ICP4 gRNAs in pX333 vector. Lanes 1 and 6, control DF1-pTIG#1 and DF1-pTIG#3 cells, respectively, transfected with empty pX333 vector. M, 1 kb + ladder. (**c**) Sequence comparison of WT and cleaved ICP4 PCR amplicons. The PAM is highlighted in red; the gRNA site is highlighted in blue and a base change is highlighted in green. (**d**) Effect of ICP4-gRNAs on MDV replication. Chicken embryo fibroblasts (CEF) were transfected with vectors carrying the Cas9 and ICP4 gRNAs (gICP4) or non-specific gRNAS (gNS) and infected with CVI988 MDV. Plaques were counted after four passages. The numbers of plaque-forming units (PFU) (on top of each column) for the cells transfected with ICP4-gRNAs (grey bar) are the average of two separate experiments and shown as the fraction of the PFU count for NS gRNAs (black bar), which is set at 1.0.

To test whether CRISPR/Cas9 can inhibit MDV replication, we selected viral ICP4 as our target gene. First, we generated three transgenic DF1 cell lines that carry ICP4 and GFP transgenes; DF1-pTIG#1, pTIG#2 and pTIG#3, respectively (Figure 1). These cell lines were used to examine CRISPR/Cas9-induced targeted cleavage at the gRNA recognition sites of the ICP4 genes. Next, we designed six gRNAs forming three pairs, which were targeted to the proximal, middle and distal parts of the viral ICP4 gene (Figure 2a), and each pair was cloned into the pX333 vector. DF1-pTIG cells were each transfected with the corresponding pX333 vector carrying gRNAs and a gGFP vector targeting the GFP gene. The empty pX333 vector was also transfected into each cell line and served as a negative control. The ICP4-targeted cells were sorted based on loss of GFP at 14 days post-transfection. The targeted Cas9 nuclease activity was validated by the production of cleaved ICP4 PCR products of the expected size in the transfected cell lines. PCR screening across the targeted regions of ICP4 fragments produced the expected WT size fragments and smaller fragments because of cleavage. As shown in Figure 2b, lanes 2, 3, 4 and 5 produced a modified PCR product resulting from CRISPR/Cas9 cleavage and the WT PCR product, confirming the targeted cleavage of ICP4 fragments in DF1_pTIG cells by corresponding ICP4 gRNAs. In contrast, only a WT PCR amplicon was produced in DF1-pTIG cell lines when these cells were transfected with the empty vector (Figure 2b, lanes 1 and 6). Unfortunately, the control DF1_pTIG#2 cells were terminated due to contamination. Targeted deletions in the smaller fragments were confirmed by Sanger sequencing (Figure 2c).

To examine the CRISPR/Cas9-mediated interference on MDV replication, we constructed three vectors. Vector 1 contains the Cas9 gene and the GFP gene under the control of the CMV-IE and CAGG promoters, respectively. Vector 2 consists of gRNA1, gRNA4 and gRNA6 (Table 1), using hU6 promoters to drive expression of the three gRNAs and the DsRed gene under the control of the CMV-IE promoter. Vector 3 contains non-specific (NS) control gRNAs and DsRed under the control of the CMV-IE promoter and served as a negative control (Figure S1). The effect on MDV replication was examined in CEF transfected with vectors 1 + 2 (Cas9 and gICP4), respectively, or vectors 1 + 3 (Cas9 and gNS) (Figure S1). Transfected CEF that were positive for GFP and DsRed were sorted using FACS and seeded into wells, followed by infection with CVI988. We performed three passages onto freshly transfected CEF and plaques were counted at the end of the fourth passage. CEF transfected with Cas9+gICP4 (no. of plaques: 12, 8) showed reduced numbers of PFU compared to Cas9+gNS (no. of plaques: 21, 15) (Figure 2d). Although the number of plaques was reduced, the sizes of the plaques did not differ between the treatment groups.

### 3.2. Generation of Transgenic Chickens Expressing gICP4 and Cas9

**Figure 3:**
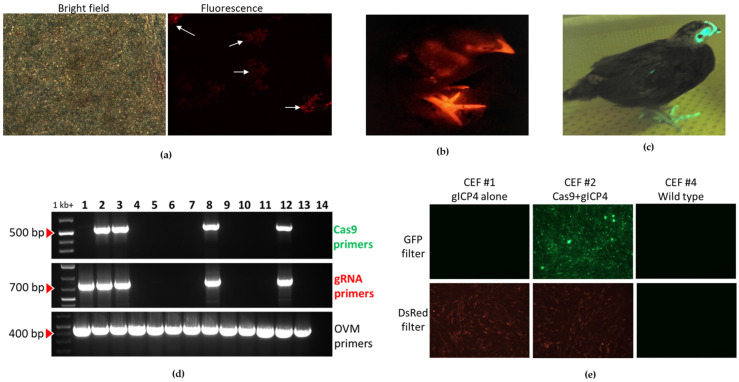
Generation of transgenic chickens and characterization of Cas9/gICP4 CEF. (**a**) Representative images of gonads harvested at stage 40 Hamburger Hamilton (HH) of injected embryos with the Tol2-gICP4-DsRed and pTrans complexed with L2000 CD complex. DsRed (right) and corresponding bright field (left) images of representative gonads. Fluorescent image of a hatched G1 gICP4-DsRed (**b**) and the Cas9-GFP (**c**) hen used to expand the Cas9-GFP line for this study. Hatched chicks were visually screened for GFP and DsRed expression using specific light sources for GFP or DsRed equipped in the goggles (BLS LTD, Hungary) and images were captured with a camera. (**d**) CEF were prepared from 10-day-old embryos obtained by breeding gICP4-DsRed and Cas9-GFP chickens. PCR genotyping of individual CEF cultures was performed using specific primers targeting the Cas9 transgene (top panel), gICP4 transgene (middle panel) or endogenous ovomucoid (OVM) gene (lower panel). Lane 1, gICP4 alone; lanes 2, 3, 8 and 12, Cas9/gICP4; lanes 4, 5, 6, 7, 9, 10, 11 and 13, WT; lane 14, water control. (**e**) Fluorescent microscopy images of CEF cultures #1, #2 and #4 expressing DsRed alone (gICP4 transgene alone), GFP+DsRed (Cas9/gICP4 transgenes) or no fluorescence (WT), respectively.

To evaluate the CRISPR/Cas9-mediated suppression of MDV replication in vivo, constructs encoding gICP4 and Cas9 were required to be introduced into the chicken germline. To generate transgenic G1 gICP4-DsRed chickens, we injected chicken embryos intravenously at stage 14–15 HH with the L2000CD-complexed Tol2-gICP4-DsRed and pTransposase vectors. Prior to hatching G0 chickens, we performed a toxicity assay by injecting embryos, and the gonads from the injected embryos were examined at stage 40 HH. Nine of the 12 injected embryos showed fluorescence (Figure 3a), indicating that stable integration of Tol2-gICP4-DsRed transgene insert and expression of three ICP4 gRNAs and DsRed were tolerated by PGCs. We then performed an injection of Tol2-gICP4-DsRed and pTransposase vectors complexed with L2000CD into 92 stage-14–15 HH chicken embryos. We raised 12 G0 chimeras to sexual maturity and their semen was assessed for the presence of gICP4-DsRed transgene using qPCR analysis. We found two promising founder roosters with the highest levels of Tol2-gICP4-DsRed transgene in semen samples (Table 2). These founder roosters (Rooster ID: #165 and #179) were each bred with 7–10 hens to generate G1 transgenic chickens. The G1 hatched chicks were screened for the presence of the transgene for DsRed expression by fluorescence. A total of 689 G1 offspring were hatched, of which 12 chickens were positive for the gICP4-DsRed transgene (Figure 3b). The germline transmission rates of individual roosters are provided in Table 2. Of the 12 G1 transgenic chickens, 3 males (Rooster ID: #278, #285 and #289) and 4 females (Hen ID: #276, #281, #287 and #290) were raised to sexual maturity and used to produce experimental embryos and chickens for in vitro and in vivo studies. The G1 transgenic gICP4-DsRed chicken identification, parental information, transgene copy number and insertional site analysis details are provided in Table 3.

Our laboratory previously generated a transgenic Cas9 hen that constitutively expresses gRNAs targeting an endogenous gene, Cas9-HF1 and GFP (Figure 3c) [30]. To expand this line by generating G2 progeny, we crossed the heterozygote G1 Cas9 hen with a WT rooster and generated a total of 21 offspring (8 males and 13 females) for further breeding. Prior to making the decision to use this Cas9-GFP chicken for in vivo work, MDV infection studies on CEF derived from this line revealed no significant differences in MDV replication between Cas9 and WT CEF (Figure S2).

### 3.3. Inhibition of MDV Replication in Transgenic CEF Expressing Cas9/gICP4

**Figure 4:**
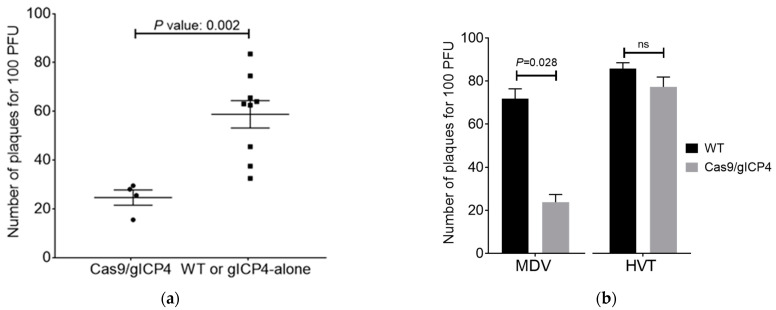
Specific inhibition of MDV and not herpesvirus of turkeys (HVT) in transgenic CEF. (**a**) Individual CEF cultures, Test (4 Cas9/gICP4) and control (total 9 CEF; 8 WT and 1 gRNA alone), were infected with 100 PFU of MDV Woodland (p19). Plaque numbers were calculated at 5 days post-infection (dpi). Error bars correspond to standard error of the mean (SEM). Statistical difference was calculated using a two-tailed Mann–Whitney test. *p* value indicates the level of significance between two groups. (**b**) The effect of the serotype 1-specific ICP4-gRNAs and Cas9 on MDV. CEF were prepared from WT (black bars) or Cas9/gICP4 (grey bars) embryos and plated at 2 × 10^6^ cells per well in a six-well plate. Triplicate cultures were infected with 100 PFU of serotype 1 MDV Woodland (p19) or serotype 3 HVT vaccine. The plaques were counted at 5 dpi. Values are shown as the mean of four replicates. Error bars correspond to SEM. *p* value indicates the level of significance between two groups. ns denotes not significant.

To test whether the Cas9 and gICP4 transgenes that were introduced into the germline were functional and mediate inhibition of MDV replication, we established primary CEF cultures, which were derived from individual embryos obtained by breeding a gICP4-DsRed rooster with a single Cas9-GFP hen. Genotyping identified four embryos as positive for Cas9/gICP4 (Figure 3d, lanes 2, 3, 8 and 12), one embryo positive for gICP4 alone (Figure 3d, lane 1) and eight embryos were double negative (Figure 3d, lanes 4–7, 9–11 and 13). To further confirm the genotypes based on fluorescence, CEF cultures were examined under a fluorescence microscope for the presence of GFP and/or DsRed. The fluorescence examination confirmed the results of the PCR assay (see Figure 3e for selected examples), showing that the germline-introduced Cas9-GFP and gICP4-DsRed transgenes were transcribed and translated for GFP and DsRed. Infection of the CEF cultures with 100 PFU of the Woodland vv MDV strain (p19) showed a significant inhibition of MDV replication in the cultures expressing GFP and DsRed (average number of PFU was 24) compared with the results for the cultures lacking expression of DsRed (average number of PFU was 58) (*p* = 0.002) (Figure 4a). These results clearly show that not only DsRed was expressed but also one or more of the gRNAs against ICP4 and that the combination of Cas9-GFP and gICP4-DsRed transgenes inhibited MDV replication.

### 3.4. ICP4 gRNAs Exhibited Virus-Specific Virus Inhibition in Cas9/gICP4 CEF

Next, we examined whether the designed ICP4 gRNAs exhibited sequence-specific inhibition of serotype 1 of MDV but not HVT, which is widely used as a vaccine for the control of MDV. To assess this, WT and Cas9/gICP4 CEF were infected with 100 PFU of MDV Woodland strain (p19) or HVT. MDV Woodland (p19) replication was significantly reduced (*p* = 0.028) in Cas9/gICP4 CEF compared with WT CEF, confirming that CRISPR/Cas9 was interfering with MDV replication. In contrast, HVT plaque numbers were similar in Cas9/gICP4 CEF and WT CEF (Figure 4b). These results show that the ICP4 gRNAs are specific for MDV ICP4 and do not interfere with HVT.

### 3.5. Inhibition of MDV Replication in Transgenic Chickens Expressing Cas9/gICP4

Following the demonstration of MDV inhibition in CEF expressing Cas9/gICP4, we next examined whether transgenic chickens expressing Cas9/gICP4 have increased resistance to MDV infection compared to WT chickens. To generate offspring carrying both Cas9/gICP4, we crossed gICP4-DsRed roosters (ID: #278, #285 and #289) with 4–10 Cas9 hens or one Cas9-GFP rooster to four gICP4-DsRed hens (ID: #276, #281, #287 and #290). Offspring from these crosses were expected to show the following genotypes: GFP alone (Cas9), DsRed alone (gICP4), GFP+DsRed (Cas9/gICP4) or WT. We compared susceptibility to MDV infection between WT or Cas9 to Cas9/gICP4 genotypes.

**Figure 5:**
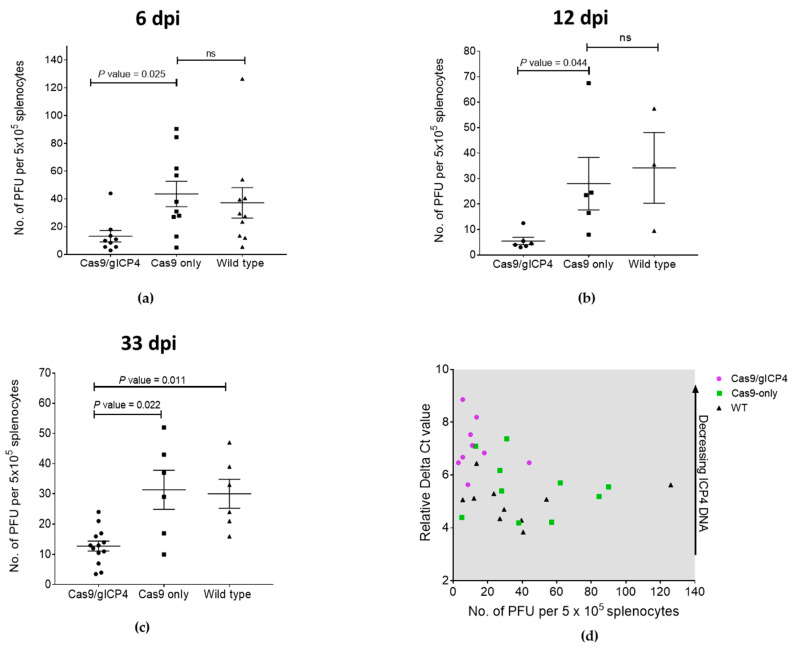
Inhibition of MDV in transgenic chickens expressing Cas9/gICP4. Transgenic chickens expressing Cas9/gICP4, Cas9 only or WT chickens were intraabdominally inoculated with MDV Woodland strain (p19). Spleens were harvested at (**a**) 6 and (**b**) 12 days post-infection (dpi) in experiment 1 and at (**c**) 33 dpi in experiment 2 for MDV isolation. Virus isolation assays were performed on splenocytes isolated from individual spleens by inoculating CEF with 5 × 10^5^ splenocytes/well in duplicate wells. PFU were counted at 5 dpi. The viral titers were shown as number of PFU per 5 × 10^5^ splenocytes. Error bars correspond to SEM. *p* value indicates the significance between two groups. ns denotes not significant. (**d**) qPCR-based verification of ICP4 DNA levels on 6-dpi experimental group chickens. *y*-axis: Delta Ct value; *x*-axis: PFU per 5 × 10^5^ splenocytes. Black dots: WT; green dots: Cas9-only; purple dots: Cas9/gICP4.

In experiment 1, two independent trials were performed. Virus replication was assessed at 6 dpi (trial 1) and at 6 and 12 dpi (trial 2). Due to the low numbers of birds available for each genotype and the absence of significant differences between the results of the control groups, the data for 6 dpi were pooled for the final analysis. Experiment 1 used a combined 15 Cas9/gICP4, 15 Cas9-only and 13 WT chicks which were intraabdominally inoculated with 2000 PFU of MDV Woodland (p19) at 7 days of age. There was no difference in virus isolation between the two control groups at 6 and 12 dpi (Figure 5a,b). However, virus titers were significantly lower in the Cas9/gICP4 group than in the Cas9 control group at 6 dpi (*p* = 0.025) and at 12 dpi (*p* = 0.044) (Figure 5a,b). In experiment 1, statistical significance was achieved between the Cas9/gICP4 and Cas9-only groups, but not the Cas9/gICP4 and WT groups, because only three chickens were available in the WT group. In experiment 2, 13 Cas9/gICP4, 6 Cas9-only and 6 WT chicks were inoculated with 2430 PFU of MDV Woodland (p19) at 7 days of age, and virus isolation rates were examined at 33 dpi. Virus isolation was significantly lower in the Cas9/gICP4 chickens than in the control groups of Cas9-only and WT chickens (*p* = 0.022 and *p* = 0.011, respectively) (Figure 5c). No significant difference was found in viral titers between the WT and Cas9-only groups. Using qPCR, we verified that the levels of viral ICP4 DNA were lower in the Cas9/gICP4 chickens than in the Cas9-only or WT chickens from the 6-dpi experimental chickens. The qPCR data showed reduced viral ICP4 levels in Cas9/gICP4 compared to Cas9-only or WT chickens and confirmed the virus titers data obtained in the virus isolation (Figure 5d).

## 4. Discussion

The present work demonstrates that the bacterial CRISPR/Cas immune system can be adapted into chickens as a novel antiviral approach. Our data show that CRISPR/Cas9 can reduce MDV replication in transgenic chickens expressing Cas9 and gRNAs programmed against the MDV ICP4 gene. The results obtained in this study present new opportunities to increase disease resistance in chickens from evolving strains of MDV and other avian pathogens with economic significance.

Expression of ICP4, an immediate early gene product, is essential for the replication cycle of herpesviruses [34,35]. Hence, we selected ICP4 as the target gene for CRISPR/Cas9-mediated virus inhibition studies. Because not all designed gRNAs exhibit successful targeting, we designed and characterized six gRNAs against ICP4, of which three selected gRNAs showed an antiviral effect when expressed along with Cas9 (Figure 2d). Our in vitro inhibition studies indicate that MDV DNA is indeed accessible for the Cas9/gRNA complex whilst multiple DSBs within the viral ICP4 alone are sufficient to inhibit MDV replication in CEF. Although the number of plaques was reduced, the sizes of plaques did not differ between the treatment groups. These results agree with previously reported RNAi-mediated targeting of ICP4 to inhibit virus replication [12,13,14]. The absence of complete inhibition of MDV replication is most likely due to one or more of the following reasons: (i) the fact that ICP4 has two copies and targeting of both copies might have not be achieved in transfected CEF; (ii) the integration of the MDV into the genome could make the targeting of MDV tricky by CRISPR/Cas9-mediated targeting, especially on an immediate early gene. A recent study has shown that the CRISPR/Cas9 system can abrogate MDV replication in vitro using a single or multiple gRNAs targeting essential genes of MDV replication. The results showed that MDV replication can not only be abrogated but can also prevent escape mutants by simultaneous targeting of gB and DNA polymerase or ICP4 genes [23]. The Cas9-mediated targeted deletions range from 1 to ~20 nt or more, which induces frameshift mutations on the coding sequence of a gene and produces either truncated or non-functional proteins [18,20,21,22,23,30]. Because we used three gRNAs that cover the entire ICP4 gene, our targeting approach will have two possible results: (i) targeted deletion of the entire ICP4 gene or (ii) mutations at three ICP4 gRNA sites. We expect that the possibility of developing mutant viruses escaping the CRISPR/Cas9-induced inhibition will be highly unlikely because it would require mutations at three locations within ICP4 at the same time in transgenic chickens, but this needs to be tested. Similar observations were found for other herpesviruses targeting one or more essential genes of human cytomegalovirus or HSV-1, where gRNAs targeting two essential genes completely blocked the formation of new infectious virus, but only partial inhibition and emergence of escape mutants was observed when one essential gene was targeted [20].

Our laboratory previously generated a transgenic chicken that constitutively expresses Cas9, GFP and gRNAs targeting an endogenous gene (Figure 3c) [30]. We performed in vitro infection of Cas9 CEF and WT CEF with the MDV Woodland strain and found no significant differences in MDV replication in Cas9 and WT CEF (Figure S2). Therefore, we generated a second transgenic chicken line that constitutively expresses three gICP4s and the DsRed marker (Figure 3b). Mating of the gICP4-DsRed and Cas9-GFP lines successfully produced offspring expressing both Cas9+gICP4 needed for the experimental studies. Previously, studies on the stable expression of small RNA molecules (such as shRNAs) in mice [15] and pigs [16] showed toxicity issues due to oversaturation of the cellular miRNA pathway. This was clearly not a problem in our studies. As gRNAs are small RNA molecules from the prokaryotic CRISPR mechanism, it is unlikely that gRNAs will enter the native miRNA pathway in chicken cells, thus allowing constitutive over-expression in eukaryotic cells.

The reduced viral load in spleen cells during the lytic phase (6 dpi) and latency (12 and 33 dpi) in Cas9/gICP4 chickens compared to the Cas9-only or WT controls (Figure 5) indicates that reduced infectivity levels in these chickens were caused by the ICP4 gRNAs directed Cas9 cleavage of the MDV genome. Two possible explanations may account for the partial clearance of virus in transgenic chickens. First, the transgenic chickens were heterozygotes and perhaps the expression of Cas9 and gICP4 from a single insert in the transgenic chickens may not have the capacity to target all copies of ICP4, as efficient recruitment of Cas9 protein to the target viral ICP4 gene is dependent on the presence of high levels of ICP4 gRNAs. This can be assessed through further breeding of the heterozygote transgenic chickens to generate two copies of Cas9 and ICP4 gRNA constructs. Second, the targeting of ICP4 gene alone may not be sufficient, allowing the viral replication to succeed at lower levels in the absence of functional ICP4. Perhaps a combinatorial MDV gene-targeting approach would provide complete inhibition of virus replication compared to what we observed in our experiments.

The fact that the birds did harbor latent virus raises the question of whether reactivation of MDV under the influence of stress or infection with immunosuppressive viruses such as chicken infectious anemia virus [36,37] may lead to reduced levels of virus replication compared to WT birds. It is also not known at the current time if the transgenic chickens are resistant to tumor development. Both questions will need to be addressed in our model as well as for the approach used by Hagag et al. [23].

We confirmed the specificity of our ICP4-gRNAs to target only the serotype 1 strains of MDV by showing inhibition of MDV Woodland strain (serotype 1) replication but not of HVT in transgenic CEF expressing Cas9/gICP4, confirming sequence-specific viral interference of the CRISPR/Cas9 system against serotype 1 viruses (Figure 4b). Thus, the antiviral strategy that we developed in this study against pathogenic serotype 1 strains of MDV can be applicable without any limitation on the use of recombinant HVT vaccines.

Prior to the development of CRISPR/Cas9 technology, the application of RNAi to combat MDV in vivo in chickens was examined. One limitation observed is that some viruses can limit RNAi efficacy in eukaryotic cells by evolving viral suppressors of RNAi through long-term coevolution [38]. In contrast and of benefit is that the CRISPR/Cas9 system is an adaptive immune defense mechanism in prokaryotes, and therefore, emergence of Cas9 evasion strategies by compensatory mechanisms from eukaryotic viruses such as MDV in transgenic chickens expressing Cas9/gICP4 genes is unlikely. In addition, we believe that CRISPR/Cas9-mediated direct targeting of viral DNA for MDV suppression will be more effective compared to RNAi-mediated knockdown of viral transcripts because CRISPR/Cas9 offers direct targeting of the viral gene, prior to transcription of an mRNA substrate required for Dicer targeting.

The introduction of off-target mutations in genomes remains a consideration when applying CRISPR/Cas9. To reduce any potential off-target concerns, we carefully considered the following factors. First, we used high-fidelity Cas9 which has previously been shown to have less off-target activity in human cells relative to SpCas9-WT [39]. Second, gRNAs were selected based on the absence of homology to the chicken genome. Together, we believe these two considerations substantially reduce any potential off-target effects on the chicken genome.

## 5. Conclusions

To our knowledge, this is the first successful report describing the inhibition of MDV replication in vivo by the stable expression of Cas9 and gRNAs targeting the viral ICP4 gene for intracellular defense against MDV. This establishes a novel antiviral strategy in eukaryotes and will open new avenues for the development of chickens resistant to other avian DNA viral diseases.

## Figures and Tables

**Table 1:** List of six designed gRNAs targeting the ICP4 gene of Marek’s disease herpesvirus (MDV).

Guide RNA	Location	Sequence (5′–3′)	PAM
gRNA1 (pair 1)	−159 nt from start codon of ICP4	GGGAAGCTACGGTTCAAGTG	CGG
gRNA2 (pair 1)	29 nt from start codon of ICP4	ACAAGCTGTCGAAATCAGGC	GGG
gRNA3 (pair 2)	3324 nt from start codon of ICP4	ATTGTTCGAGCCGCCCGCGT	CGG
gRNA4 (pair 2)	3481 nt from start codon of ICP4	ATTGCGTTGGACGGCTCGGC	CGG
gRNA5 (pair 3)	6234 nt from start codon of ICP4	TCAATGATACATGGACCGTT	TGG
gRNA6 (pair 3)	6484 nt from start codon of ICP4	GTATACAGTCGAAACGCGCC	TGG

**Table 2:** Germline transmission and transgenic gICP4-DsRed chicken production.

G0 Rooster No.	Relative Levels of Tol2-gICP4-DsRed in Semen ^1^	Offspring Hatched	Transgenic Offspring	Percentage Transgenesis
179	0.62	212	5	2.36
165	0.55	477	7	1.47

^1^ The relative levels of Tol2-gICP4-DsRed transgene-integrated genomic DNA in semen was calculated by comparison of the mean Ct values of genomic and miniTol qPCR primers from three individual semen samples.

**Table 3:** Transgenic gICP4-DsRed parental information, sex and transgene copy number of gICP4-DsRed.

G0 Transgenic Parent	G1 gICP4-DsRed	Sex	Location	Chromosome	No. of Transgene Inserts
165	278	M	ND	-	1
	289	M	Intron, ETS1 (transcription factor)	24	1
	281	F	Intron, genscan prediction	8	1
	287	F	Intron, genscan prediction	1	1
179	285	M	Intron, genscan prediction	2	1
	276	F	Intron, not annotated	11	2
	290	F	Intron, not annotated	11	2

ND denotes not done.

## Data Availability

Data is contained within the article or supplementary material.

## Supplementary Materials

The following are available online at https://www.mdpi.com/2076-2607/9/1/164/s1, Figure S1: Vector 1 encoding Cas9-GFP (top), vector 2 encoding gICP4-DsRed (middle), vector 3 encoding gNS-DsRed (bottom). Figure S2: Six individual Cas9-only or WT CEF cultures were plated at 2 × 106 cells per well in duplicates and infected with 100 PFU of MDV Woodland strain (p19). Plaques were counted at 5 dpi. Error bars correspond to SEM; t-test was performed. ns denotes not significant. Figure S3: Uncropped gel images for Figure 2b. Details of the lanes in the gels are provided in figure legend 2b. Supplementary Table S1. List of primers used in the study.
