# Supplementary material for: In Vivo Inhibition of Marek’s Disease Virus in Transgenic Chickens Expressing Cas9 and gRNA against ICP4
Source: Microorganisms. 2021 Jan 13;9(1):164. doi: 10.3390/microorganisms9010164 (PMC7828426; doi:10.3390/microorganisms9010164)
Supplement: Supplementary file 1 [file microorganisms-09-00164-s001.pdf]

microorganisms

MDPI

## Supplementary information

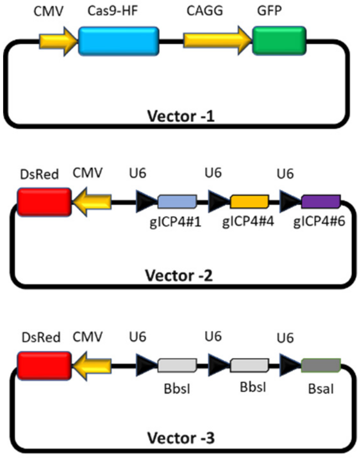


**Figure S1:** Vector 1 encoding Cas9-GFP (top), vector 2 encoding gICP4- DsRed (middle), vector 3 encoding gNS-DsRed (bottom)

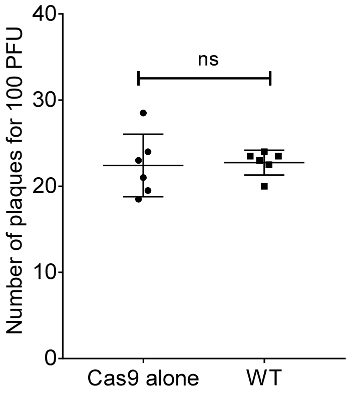


**Figure S2:** Six individual CEF cultures of Cas9-only or WT were plated at $2x10^6$ cells per well in duplicates and infected with 100 PFU of MDV Woodland strain (p19). Plaques were counted at 5 dpi. Error bars correspond to SEM. T-test was performed. ns denotes not significant.

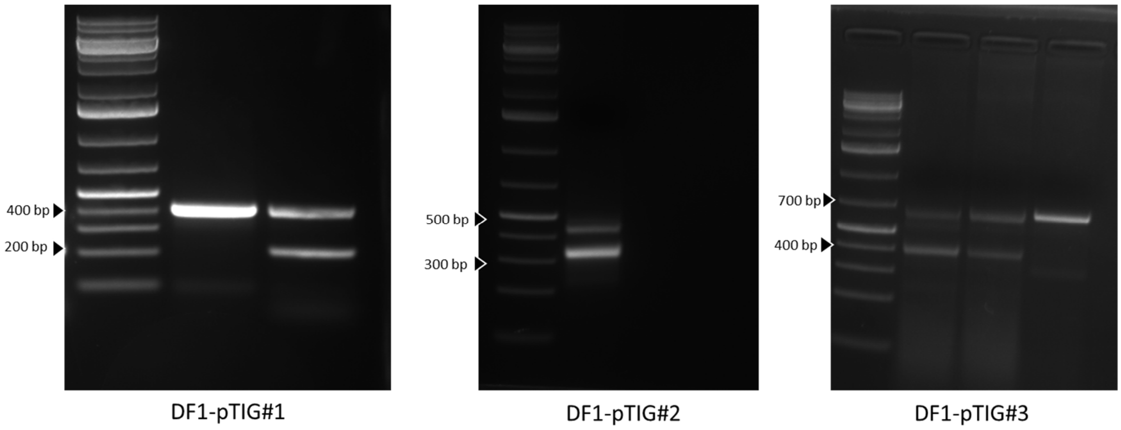


**Figure S3:** Uncropped gel images for Figure 2b. Details of the lanes in the gels were provided in figure legend 2b.

**Supplementary Table S1.** List of primers used in the study.

| PCR Primer | Use | Sequence (5′-3′) |
|---|---|---|
| gRNA_1_Fwd | PCR | CACCGGGAAGCTACGGTTCAAGTG |
| gRNA_1_Rev | PCR | AAACCACTTGAACCGTAGCTTCCC |
| gRNA_2_Fwd | PCR | CACCGACAAGCTGTCGAAATCAGGC |
| gRNA_2_Rev | PCR | AAACGCCTGATTTCGACAGCTTGTC |
| gRNA_3_Fwd | PCR | CACCGATTGTTCGAGCCGCCCGCGT |
| gRNA_3_Rev | PCR | AAACACGCGGGCGGCTCGAACAATC |
| gRNA_4_Fwd | PCR | CACCGATTGCGTTGGACGGCTCGGC |
| gRNA_4_Rev | PCR | AAACGCCGAGCCGTCCAACGCAATC |
| gRNA_5_Fwd | PCR | CACCGTCAATGATACATGGACCGTT |
| gRNA_5_Rev | PCR | AAACAACGGTCCATGTATCATTGAC |
| gRNA_6_Fwd | PCR | CACCGTATACAGTCGAAACGCGCC |
| gRNA_6_Rev | PCR | AAACGGCGCGTTTCGACTGTATAC |
| Poly-A-Fwd | PCR | ACCGGTCATCATCACCATCACCATTG |
| Poly-A-Rev | PCR | GGCCGGCCCATAGAGCCCACCGCATCCC |
| PCR-pX458-Fwd | PCR | CCCGGGGTCGACGAGGGCCTATTTCCCATGAT |
| PCR-pX458-Rev | PCR | CCCGGGCTCGAGAAAAAAGCACCGACTCGGTG |
| PCR-pX333-Fwd | PCR | CCCGGGGTCGACCGAGAATGGCGCATGTGAG |
| PCR-pX333-Rev | PCR | CCCGGGCTCGAGGGCGGGCCATTTACCGTAAG |
| DsRed-Fwd | PCR | GCGTCGACTCGCCACCTCTGACTTGAGC |
| DsRed-Rev | PCR | GCGGAATTCGTTCACGTAGTGGGCCATCG |
| ICP4-P-Fwd | PCR | ACTGCAATCGTTCAGCAGGACAC |
| ICP4-P-Rev | PCR | ACTAATCGGGATCTAGCGCCTTG |
| ICP4-M-Fwd | PCR | ACTACCGAGATTACCTGGGTGGA |
| ICP4-M-Rev | PCR | ACTGTCTGGACCACATAGGAGCG |
| ICP4-D-Fwd | PCR | ACTGCTGTTTATGGCGCGTATGG |
| ICP4-D-Rev | PCR | ACTTCATTGCCCGAATGTCCGAA |
| ICP4-#1-Fwd | PCR | AGATACGTATAACAATGCTG |
| ICP4-#1-Rev | PCR | GCAGCGAGACGCCTTGATAA |
| ICP4-#2-Fwd | PCR | CGGAACGTCGGACATGTTTC |
| ICP4-#2-Rev | PCR | TAAGTGCTTGTCGATGCCCA |
| ICP4-#3-Fwd | PCR | CTTACCCAAGAGCAGTGCGT |
| ICP4-#3-Rev | PCR | CGAGCCGTCGGATGTAAGAA |
| Cas9-Fwd | PCR | GTGCGTCAGCTCAATCGTTC |
| Cas9-Rev | PCR | TAAGTTTCCGCGACAAGGCT |
| gRNA-Scr-Fwd | PCR | CACCGGGAAGCTACGGTTCAAGTG |

| | | |
|---|---|---|
| gRNA-Scr-Fwd | PCR | AAACGGCGCGTTTCGACTGTATAC |
| OVM-Fwd | PCR | CGCGGACCTCAATAAATATTACCAGCCT |
| OVM-Rev | PCR | CGCCCACCTCGTTATGGCCTTGT |
| ICP4-qPCR-Fwd | qPCR | TCGTGCTTTCTGTGCTCGAT |
| ICP4-qPCR-Rev | qPCR | CGAGACCCCAGGACGTTCT |
| ICP4-Probe | qPCR | FAM-TGCACTGCATTCCGAGAG-TAMRA |
